# Leidenfrost levitation: beyond droplets

**DOI:** 10.1038/srep00797

**Published:** 2012-11-12

**Authors:** Ali Hashmi, Yuhao Xu, Benjamin Coder, Paul A. Osborne, Jonathon Spafford, Grant E. Michael, Gan Yu, Jie Xu

**Affiliations:** 1 Mechanical Engineering, Washington State University, Vancouver, 98686, USA; 2 These authors contributed equally to this work.

## Abstract

Friction is a major inhibitor in almost every mechanical system. Enlightened by the Leidenfrost effect – a droplet can be levitated by its own vapor layer on a sufficiently hot surface – we demonstrate for the first time that a small cart can also be levitated by Leidenfrost vapor. The levitated cart can carry certain amount of load and move frictionlessly over the hot surface. The maximum load that the cart can carry is experimentally tested over a range of surface temperatures. We show that the levitated cart can be propelled not only by gravitational force over a slanted flat surface, but also self-propelled over a ratchet shaped horizontal surface. In the end, we experimentally tested water consumption rate for sustaining the levitated cart, and compared the results to theoretical calculations. If perfected, this frictionless Leidenfrost cart could be used in numerous engineering applications where relative motion exists between surfaces.

Friction is a blessing in some mechanical systems whereas it is a nuisance in most. It is a blessing because it can help us stop motion, such as braking a car. However, more often friction opposes the desired motion. Approximately one-third of the world's energy resources are consumed by friction in one form or another^1^. A recent estimate shows that the annual friction-induced costs in the U.S. are roughly $270 billion to $800 billion^2^. Therefore if better lubrication technologies can be developed, enormous savings could be achieved. A novel way to reduce friction is by levitation, such as Maglev trains. One simple but elegant levitation method that has been long overlooked is Leidenfrost levitation; more than 250 years ago, J. G. Leidenfrost reported that water droplets can be levitated if placed on a red-hot spoon^3^. (Another saying is that Boerhaave reported this phenomenon 280 years ago, but his work is no longer accessible^4^.) These droplets are highly mobile and they hover around on the hot surface, experiencing negligible friction. It has been understood later that these droplets are supported by their own vapor and this phenomenon corresponds to the film boiling state of a droplet^5^. Leidenfrost levitation only happens when the substrate temperature is above the Leidenfrost point. Over the years, several theoretical models have been developed to explain and predict the Leidenfrost point; however, none of them is accurate enough to effectively capture all the possible Leidenfrost mechanisms^6^. In recent years, significant progresses have been made on controlling Leidenfrost levitation, such as self-propelled Leidenfrost droplets^7^,^8^,^9^, no-transition-state Leidenfrost phenomena^10^,^11^, and destabilization of Leidenfrost vapor by charging the droplets^12^. However, most of these studies focused on liquid droplets. We believe that Leidenfrost levitation has great potential in practical applications if we explore this phenomenon beyond droplets. In this paper, we demonstrate for the first time that Leidenfrost levitation can potentially be harnessed for levitating mechanical solid parts, which is referred to later as “Leidenfrost cart”, for frictionless motion. Several important factors affecting Leidenfrost cart have been carefully characterized and explained in following sections.

## Results

### Maximum load

**Figure 1:**
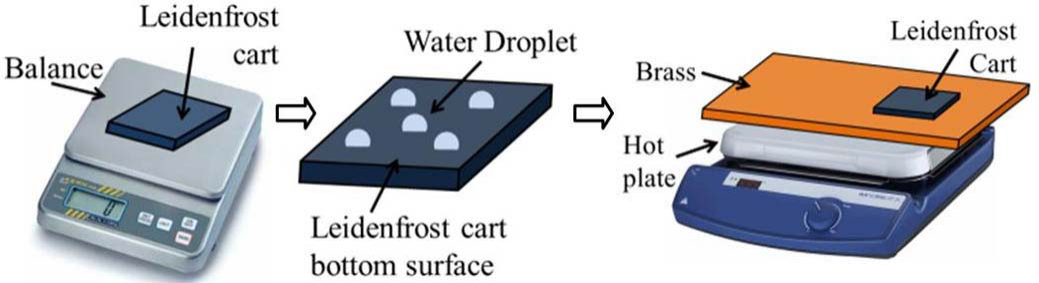
Experimental setup and methods. A cart is first weighed and then picked up for water addition onto its bottom surface. A heated brass surface is used to generate Leidenfrost levitation on the wetted cart.

**Figure 2:**
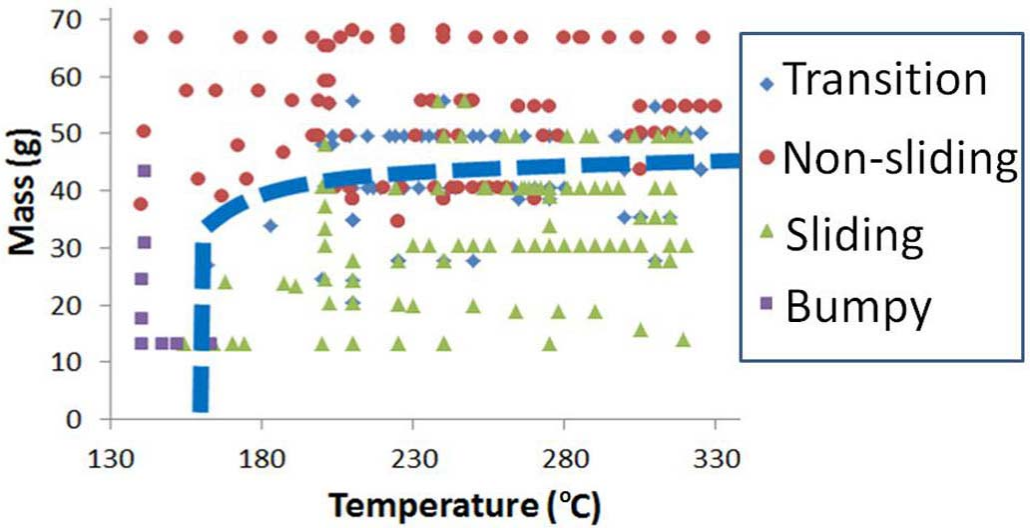
Effects of mass and surface temperature on the motion states of the Leidenfrost cart. The dashed curve separates the sliding regime from all other unwanted regimes, including non-sliding, transition and bumpy motion states.

The first set of experiments we performed aim at testing the maximum load of the Leidenfrost cart for carrying capacity at a range of surface temperatures by gradually varying the mass of the cart. A PMMA (50 mm × 50 mm × 4 mm, 10 grams) piece is used as the cart in this set of experiments. A brass substrate kept at a constant small angle of inclination θ (~2 degrees) provides the necessary conditions for the cart to move noticeably under the action of gravity. A constant volume of water 0.4 mL is added onto the bottom surface of the cart in form of small evenly dispensed droplets using a syringe as shown in Figure 1. The cart is picked up from the sides (by hand for a large cart, or with the aid of tweezers for a small cart) and is dropped vertically onto the heated substrate from a constant height of 5 mm, while ensuring that no initial horizontal velocity is imparted to the cart. It is observed that several situations may occur: sliding, non-sliding, transition, and bumpy motion, the last three being unwanted motion states. These situations are characterized with respect to surface temperature and the amount of load (total mass, including cart itself, added mass, and added water), which are reported in Figure 2.

The dashed curve separates the sliding regime from all other unwanted regimes as previously mentioned. From Figure 2, we can see that if the surface temperature is below 140°C, the cart will have a bumpy motion. This is owing to rapid bubble formation and collapse underneath the cart in the nucleate boiling regime, which is expected because the Leidenfrost point of our substrate plate is measured to be 140°C. With an increase in surface temperature we observe the cart to slide and hold a certain amount of weight up to a maximum value; beyond this weight limit, the cart does not slide freely on the surface. This is very possibly due to thinning and disruption of the Leidenfrost vapor film, causing the Leidenfrost cart to stick to the substrate. In between the sliding and the non-sliding regimes we also observe a transition region where the cart either rotates or moves momentarily. The reason for non-sliding and transition may again possibly be due to the thinning of Leidenfrost vapor film that supports the weight of the cart to a point where the vapor film is no longer stable. We postulate that the threshold mass that the cart can hold corresponds to the minimum thickness of the stable vapor film that can sustain conditions of constant levitation. Any further increase in weight might lead to a reduction of vapor film thickness resulting in a transition from non-wetting to wetting state, inducing friction and nucleate boiling under the cart. Possible mechanisms for this transition include hydrodynamic instability, metastable liquid state, thermomechanical effect and adsorption induced wetting^6^. When the cart is successfully levitated by a stable vapor film a relation between the weight *mg* and the vapor film thickness *δ* can be developed using the lubrication theory, continuity equation and the heat conduction equation, assuming the film has a uniform thickness^5^,^13^,^14^. By manipulating the equations with appropriate boundary conditions and assumptions, the maximum cart weight (*mg*) characterized as a function of film thickness can be described as: 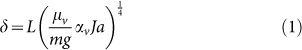
where *Ja* is the Jacob number, *α_v_* is the thermal diffusivity of the vapor, *µ_v_*is the dynamic viscosity of the vapor, *L* is the length of the square base of the cart and *δ* is the vapor film thickness. However, because a Leidenfrost cart is much larger than a Leidenfrost droplet, the experimental verification of the film thickness is challenging at this stage.

### Gravitational propulsion

To monitor whether our Leidenfrost cart moved frictionlessly we conceived a simple experiment to characterize and compare the acceleration component of the cart with the acceleration component due to gravity, g·sin(θ). The brass plate was inclined at different angles and the acceleration of the cart determined from the videos captured by the high speed camera was found to be in close agreement with the acceleration component due to gravity (see Table 1) suggesting that the cart moves almost frictionlessly. Note that without a constant supply of water the constant value of acceleration will not be maintained and the cart will eventually come to a halt. However, the length of the substrate used in our experiment (25 cm) is not significant for detecting any noticeable change in acceleration.

**Figure 3:**
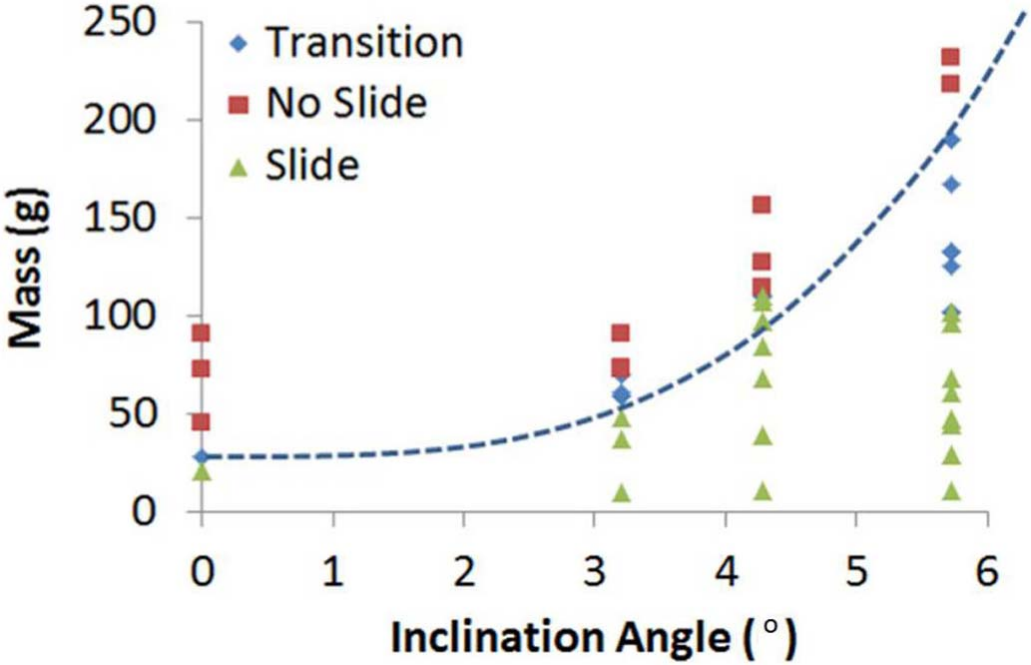
The effects of angle on the load bearing capability of the Leidenfrost cart.

Furthermore, we observe a trend between the angle of inclination and the maximum load bearing capability of the Leidenfrost cart (see Figure 3). It is anticipated that the maximum load bearing capacity for a given surface temperature will increase to an asymptote as the tilt angle approaches to 90°. The reason for the increment of the load carrying capacity at high angles of tilt is due to the reduced magnitude of the weight component (mg·cos(θ)) trying to press the cart against the incline, which is the major cause of destabilization of the supporting vapor film.

### Self-propulsion

**Figure 4:**
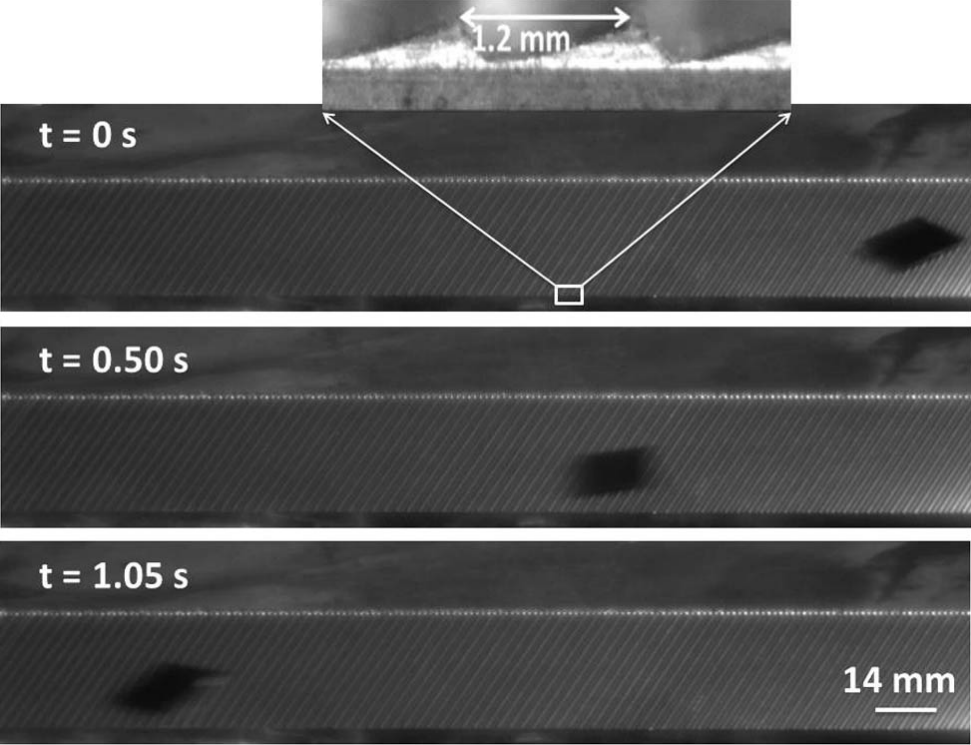
The image shows a self-propelled Leidenfrost cart on top of a horizontal ratchet shaped surface. Shown in the inset is the distance of separation between two consecutive ratchets.

Previous research has shown that water droplets on a hot ratchet will be propelled by the vapor generated between the ratchet and the droplet^7^,^8^,^9^. Here, we tested our hypothesis that Leidenfrost cart should also exhibit the ability to self-propel on a ratchet surface. In this set of experiments, a piece of silicon wafer (15.5 mm × 14 mm) is used as our Leidenfrost cart. A ratchet shape with 1.2 mm pitch and 350 μm depth (see Figure 4, inset) is used for the experiments. Figure 4 shows that our hypothesis is correct: the Leidenfrost cart can self-propel over a horizontal ratchet surface.

**Figure 5:**
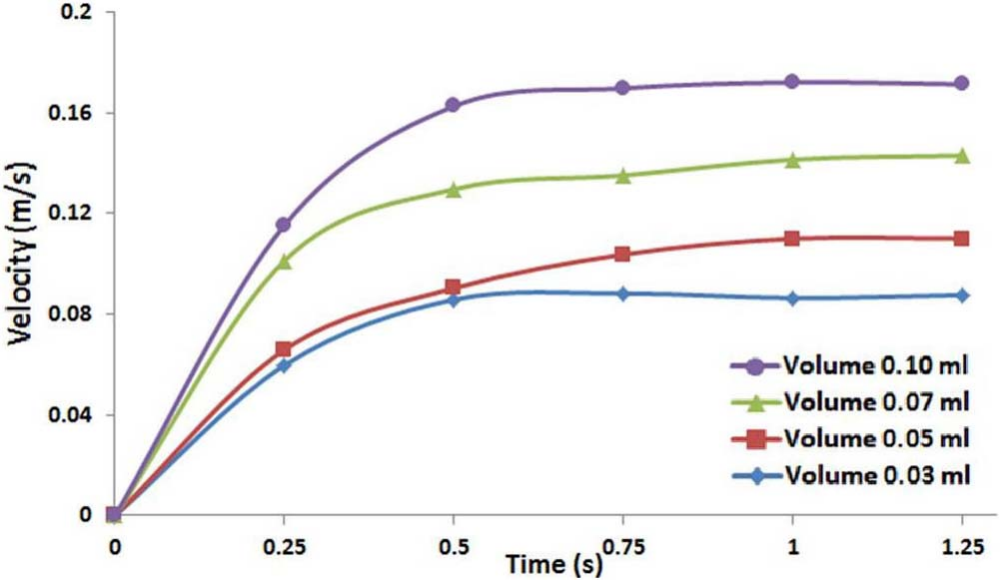
The relation between the velocity of Leidenfrost cart and the volume of initial water added.

Further experiments find that the self-propulsion of Leidenfrost cart highly depends on the amount of water under the cart. Therefore, in another set of experiments, we test the effects of initial liquid volume on the performance of the self-propelled cart. A given amount of water, ranging from 0.03 mL to 0.1 mL, is applied to the bottom of the cart and then this cart is placed on the heated ratchet surface at a constant temperature of 355°C. The cart's motion over the ratchet surface is recorded by a high speed camera. Velocity of the Leidenfrost cart is then extracted from videos and plotted in Figure 5. Note that, in all four cases, the cart accelerates at the beginning and tends to reach a constant terminal speed. We believe that if our plate is long enough the cart would eventually decelerate owing to the consumption of water through evaporation. Another observation is that the greater the initial volume of water added the faster the cart can be propelled, and water volume below 0.03 mL cannot support the cart's motion on the ratchet-shaped surface. Furthermore, experimental observations delineate that the direction of the cart's motion over the ratchet conforms to that of the individual liquid drops previously reported. Nevertheless, anomalous cases have been noticed during the experimentation on a chemically-contaminated ratchet surface. Further investigation is needed to fully understand the anomaly.

### Water consumption rate

**Figure 6:**
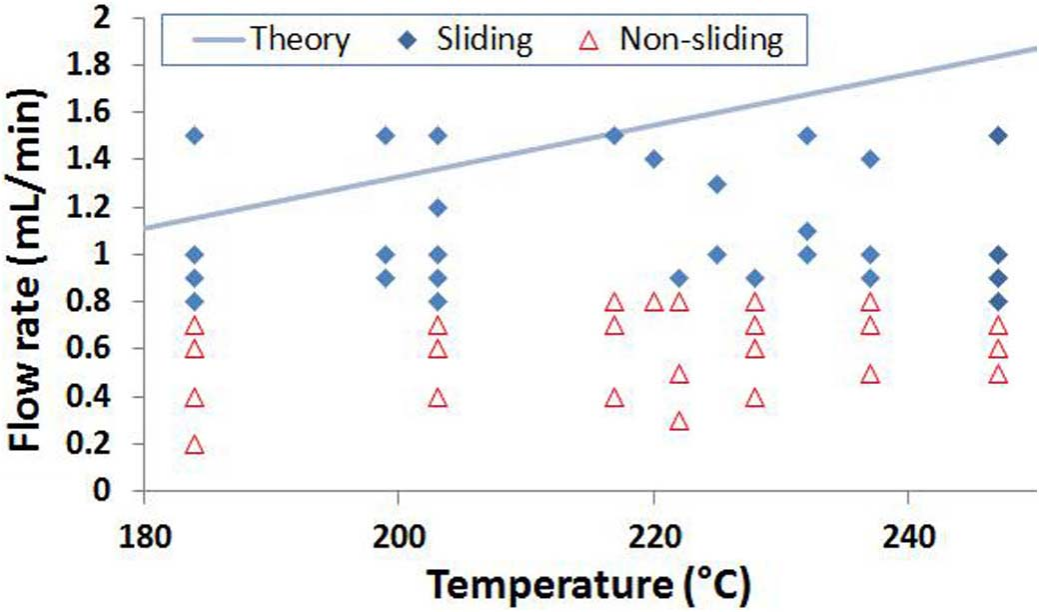
The motion states of a Leidenfrost cart fed with a constant flow rate from the centre of the cart. The cart is connected to a horizontal spring for inducing periodic motion. In a non-sliding state, the cart stops within 6 periods. In a sliding state, the cart usually oscillates for 20~40 periods before the motion is damped out. The theoretical curve indicates water consumption rate calculated from equation (2).

Biance et al^13^ measured the evaporation rate of a Leidenfrost drop by maintaining a stationary Leidenfrost droplet using constant water feeding into the droplet. Enlightened from this work, in our last set of experiments, we determine the minimum possible flow rate that could maintain conditions of constant levitation for the Leidenfrost cart at a given surface temperature. For this purpose, a layer of PDMS was spin-coated on top of a thin aluminium disc with a hole drilled in the centre to ensure inlet for water. PDMS is spin coated onto the aluminium disc to prevent the disc surface from attaining high temperatures. A needle is inserted through the disc, attached on one end to an adjustable syringe pump (KDS 210). Before conducting the experiments, the brass plate is heated to achieve a steady-state temperature. The aluminium-PDMS composite disc (12.22 g) is placed on top of the heated substrate with the PDMS side facing the hot-plate. The flow-rate is adjusted until the disc just begins to slide, hovering on top of the substrate with almost no friction to hamper its motion. In order to regulate this “hovering” observation for more quantitative assessment, a spring is attached to the cart horizontally, so that the cart can oscillate about a fixed location. Repeated experiments are performed at different temperatures as well as water flow rates, and the results are reported in Figure 6. A flow-rate below a threshold value (~0.8 mL/min) could yield a cart oscillation either momentary or only over a few periods (< 6) with obvious frictional effects. The reason may be due to dry-out of water-film underneath the disc, and since replenishment is slow under low flow-rate conditions the frictional effects become dominant. In contrast, a flow-rate above a threshold value (~0.8 mL/min) normally yields a cart oscillation over 20~40 periods before the motion is damped out. We believe the threshold value is such where we achieve a dynamic equilibrium between the rate of evaporation and the inflow. An analytical model is developed to determine the in-flow rate for conditions of constant levitation, the results of which are compared with the experimental results. By following a similar procedure as used for deriving equation (1) but now in the polar coordinate system and assuming heat conduction to be the major heat transfer mode for the evaporation of water, the minimum flow-rate (*dV/dt*) of water necessary to sustain constant levitation can be derived as: 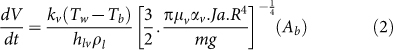
where *k_v_* is the thermal conductivity of the vapor, *T_w_* is the substrate temperature, *T_b_* is the base temperature of the Leidenfrost cart, *h_lv_* is the latent heat of vaporization, *ρ_l_* is the density of liquid and *A_b_* is the liquid film area, which is assumed to be the same as the base area of the cart (with radius, *R*, equal to 3.5 cm).

It is to be noted that the theoretical estimate of the water consumption rate is found to be higher than the experimentally determined inflow. The possible reason is that the liquid film area is over-estimated in equation (2). Indeed, with a transparent Leidenfrost cart, we observed that injected water tends to form random fingers under the cart instead of covering the entire bottom surface of the cart. Therefore, optimization of a liquid injection system using multiple inlets is recommended for future application of the Leidenfrost cart.

## Discussion

In summary, we have demonstrated, characterized and explained a novel levitation method using Leidenfrost vapor. The maximum load of the Leidenfrost cart has been explored. Different methods of propulsion have been tested. Water consumption rate has been measured and calculated. The current method being employed consumes a lot of energy for heating because of a high Leidenfrost point. On the other hand, using liquids with very low Leidenfrost point (e.g. cryogenic liquids) is also not energetically feasible because of the production cost. A near room temperature Leidenfrost point is desired for future application. This is possible with surface modification as suggested by a recent study^11^. In entirety, if perfected, Leidenfrost levitation and self-propulsion could open up a wide range of new applications.

## Methods

The experimental setup and method used in this study are very simple. A brass plate is heated by a hotplate, and the Leidenfrost point for this specific plate is tested to be around 140°C, meaning droplets will be levitated if the surface temperature is above 140°C. A cart is made of a small piece of solid with a flat surface, such as PMMA, Silicon, and PDMS-aluminum composite. In the experiments, after the weight of the cart is measured, we dispense an array of water droplets on the bottom of the cart, and then we flip the cart and place it on the hot brass surface. Separate experiments were set up to study the motion of the cart under various conditions. A high speed camera (Pixelink) is used to capture videos for quantitative analysis. The ratchet surface (1.2 mm pitch and 350 μm depth) is milled out on a brass surface using Minitech micro milling machine by tilting the spindle to a 15 degree angle.

## Author Contributions

BC and PO conceived the initial idea of this research. GY made Figure 1. BC, PO, JS and GM demonstrated the initial idea and collected data for Figure 2. JS and PO machined the Leidenfrost carts. JX, AH and YX machined the ratcheted surfaces. AH and YX collected data for Figures 3,4,5,6 with GY. AH and PO performed data analysis, and explained the physics behind the results. AH and JX wrote the paper. All authors participated in the discussions throughout the work. JX guided the work.

## Figures and Tables

**Table 1:** Acceleration of Leidenfrost cart on inclined hot surface

θ (°)	4.2±0.5	8.2±0.5	11.9±0.5
g·sin(θ) (m/s^2^)	0.72±0.08	1.40±0.08	2.03±0.08
a (m/s^2^)	0.74	1.41	2.13
